# Fine root tradeoffs between nitrogen concentration and xylem vessel traits preclude unified whole‐plant resource strategies in *Helianthus*

**DOI:** 10.1002/ece3.1947

**Published:** 2016-01-20

**Authors:** Alan W. Bowsher, Chase M. Mason, Eric W. Goolsby, Lisa A. Donovan

**Affiliations:** ^1^ Department of Plant BiologyUniversity of Georgia2502 Miller Plant SciencesAthensGeorgia30602; ^2^ Interdisciplinary Toxicology ProgramUniversity of GeorgiaAthensGeorgia

**Keywords:** Resource use, root anatomy, root nitrogen, root tissue density, specific root length, trait evolution

## Abstract

Recent work suggests variation in plant growth strategies is governed by a tradeoff in resource acquisition and use, ranging from a rapid resource acquisition strategy to a resource‐conservative strategy. While evidence for this tradeoff has been found in leaves, knowledge of root trait strategies, and whether they reflect adaptive differentiation across environments, is limited. In the greenhouse, we investigated variation in fine root morphology (specific root length and tissue density), chemistry (nitrogen concentration and carbon:nitrogen), and anatomy (root cross‐sectional traits) in populations of 26 *Helianthus* species and sister *Phoebanthus tenuifolius*. We also compared root trait variation in this study with leaf trait variation previously reported in a parallel study of these populations. Root traits varied widely and exhibited little phylogenetic signal, suggesting high evolutionary lability. Specific root length and root tissue density were weakly negatively correlated, but neither was associated with root nitrogen, providing little support for a single axis of root trait covariation. Correlations between traits measured in the greenhouse and native site characteristics were generally weak, suggesting a variety of equally viable root trait combinations exist within and across environments. However, high root nitrogen was associated with lower xylem vessel number and cross‐sectional area, suggesting a tradeoff between nutrient investment and water transport capacity. This led to correlations between root and leaf traits that were not always consistent with an acquisition–conservation tradeoff at the whole‐plant level. Given that roots must balance acquisition of water and nutrients with functions like anchorage, exudation, and microbial symbioses, the varied evidence for root trait covariation likely reflects the complexity of interacting selection pressures belowground. Similarly, the lack of evidence for a single acquisition–conservation tradeoff at the whole‐plant level likely reflects the vastly different selection pressures shaping roots and leaves, and the resources they are optimized to obtain.

## Introduction

Understanding trait variation across species and its relevance to environmental adaptation are two of the major goals of plant physiological ecology. Over the last several decades, both theoretical and experimental work have described a global pattern of covariation in leaf traits, ranging from species that produce leaves with high maximum photosynthetic rates and high‐nutrient concentrations, but low structural investment and short life span, to those exhibiting lower photosynthetic rates and nutrient concentrations, but higher structural investment and longer life span (Grime 1977; Chapin 1980; Reich et al. 1997; Wright et al. 2004). This pattern of leaf trait covariation is referred to as the leaf economics spectrum, as it summarizes investment and returns in resource‐use traits, and suggests a tradeoff between rapid resource (e.g., nutrients and water) acquisition and resource conservation in higher plants (Reich et al. 1997; Wright et al. 2004). Evidence suggests that the strategies at the opposite ends of this resource acquisition–conservation tradeoff axis are broadly adaptive in resource‐rich versus resource‐poor environments, respectively (Cunningham et al. 1999; Wright and Westoby 1999; Ordonez et al. 2009; Reich 2014).

Fine roots (the most distal branches of the root system) are often considered functionally analogous to leaves in that both tissues are relatively short‐lived and mainly involved in resource acquisition (Eissenstat and Yanai 1997). Given that belowground resources are needed for functioning of aboveground tissues (and vice versa), fine root trait covariation has been hypothesized to reflect the tradeoff described above for leaves, resulting in a single axis of resource‐use strategies (from rapid resource acquisition to greater resource conservation) at the whole‐plant level (Eissenstat and Yanai 1997; Withington et al. 2006; Freschet et al. 2010; Liu et al. 2010; Kembel and Cahill 2011; Mommer and Weemstra 2012; Reich 2014). Thin roots with high specific root length (SRL; cm·g^−1^), low root tissue density (RTD; g·cm^−3^), and high root nitrogen concentrations (root N; g·g^−1^) are generally expected to represent the “resource acquisition” end of the spectrum, and to exhibit rapid elongation (Eissenstat 1991) and nutrient uptake rates (Comas et al. 2002), high hydraulic conductivity (Solari et al. 2006), potentially due to large diameter xylem vessels which may be particularly important for water transport (Chiu and Ewers 1992; Hargrave et al. 1994), and short root life span (Tjoelker et al. 2005). Thick roots with high RTD, low SRL, and low root N represent the “resource conservation” end of the spectrum and are generally assumed to exhibit lower resource uptake capacity, slower growth rates, and longer life span (Eissenstat et al. 2000; Mommer and Weemstra 2012; Kong et al. 2014), due in part to a large proportional investment in tough secondary cell walls (i.e., high number of xylem vessels per root cross‐sectional area, high total xylem cross‐sectional area, high proportion of root CSA in xylem and/or stele; Wahl and Ryser 2000; Hummel et al. 2007). As with leaves, these contrasting strategies of maximizing resource acquisition versus resource conservation are hypothesized to be broadly adaptive in high‐ versus low‐resource environments, respectively (Fort et al. 2012). However, empirical studies have found mixed support for these expected patterns in fine root traits and their relation to resource availability (Espeleta and Donovan 2002; Nicotra et al. 2002; Tjoelker et al. 2005; Comas and Eissenstat 2009; Kembel and Cahill 2011; Fort et al. 2012; McCormack et al. 2012; Chen et al. 2013; Kong et al. 2014). As a result, our understanding of the evolution of root trait strategies across species and environments is limited in comparison with leaves (Chen et al. 2013; Kong et al. 2014; Reich 2014).

The lack of agreement among studies of root trait covariation across species and environments could stem from several possible factors. First is the potentially confounding influence of environmental gradients, as large‐scale studies (>25 species) assessing root trait variation have largely been conducted in the field (e.g., Craine et al. 2001; Chen et al. 2013; Comas et al. 2014; Kong et al. 2014; but see Wright and Westoby 1999). Thus, patterns of trait covariation and their relationships with resource availability observed in such studies might be due, at least in part, to plastic responses to environmental gradients (Rausher 1992; Reich et al. 2003). Common garden studies, on the other hand, allow for determination of genetically based differences among species and interpretation of how these differences may reflect adaptive differentiation among species (Wright and Westoby 1999; Nicotra et al. 2002).

Second, selection to simultaneously optimize the numerous functions of roots may preclude the evolution of a single axis of trait strategies that reflects adaptation to resource‐rich versus resource‐poor environments (Comas et al. 2012; Chen et al. 2013; Kong et al. 2014). No matter the environment, roots must balance acquisition of both water and nutrients, as well as other functions such as root exudation, anchorage, and support of mycorrhizal fungi (Brundrett 2002; Comas et al. 2012). Given that a variety of root traits may relate to these functions, many different combinations of root traits may be equally successful in similar environmental conditions (Comas et al. 2012). The opposite may also be true: Similar combinations of root traits may be equally successful in different environmental conditions (Ryser 2006). For example, high SRL allows for rapid root elongation and maximum surface area for nutrient uptake, both of which likely benefit competitive species typical of fertile habitats. However, high root surface area resulting from high SRL may also benefit plants from infertile habitats by allowing for increased acquisition of limiting soil resources (Ryser 2006; Zangaro et al. 2008; Holdaway et al. 2011). Analogous scenarios for other root traits such as RTD and root N may have contributed to the general lack of agreement among studies examining trait covariation and its relation to environmental resource availability. Additional investigations of species distributed across environmental gradients could help establish whether fine root trait variation, and the anatomical traits underlying that variation, can be generalized into tradeoff spectra (Kong et al. 2014).

A third possible explanation for the lack of agreement among studies of root trait covariation is the influence of species' evolutionary relationships (Felsenstein 1985; Harvey and Purvis 1991). Because of their hierarchical relatedness, closely related species often have a tendency to resemble one another more closely in trait values than more distantly related species; a condition called phylogenetic signal (Abouheif 1999; Blomberg and Garland 2002). In situations with high phylogenetic signal, species cannot be viewed as statistically independent data points, violating a main assumption of correlation analyses often used to assess trait covariation (Felsenstein 1985; Harvey and Purvis 1991). A number of methods for accounting for phylogeny in investigations of trait covariation exist (Felsenstein 1985; Grafen 1989; Housworth et al. 2004). However, the use of such methods in the absence of phylogenetic signal (Blomberg et al. 2003; Rheindt et al. 2004; Garland et al. 2005), or more specifically in the absence of phylogenetic signal in the residual error of the regression model (Revell 2010), is inappropriate, and can even produce artifactual results due to violations of the evolutionary models implicit in phylogenetic comparative methods (Abouheif 1999; Rheindt et al. 2004; Revell 2010). Therefore, assessment of levels of phylogenetic signal is essential in comparative studies examining trait covariation (Gittleman and Luh 1992; Blomberg et al. 2003).

In this study, our broad objective was to investigate evolutionary patterns in fine root trait variation and test whether these patterns provide evidence for adaptive differentiation in relation to native site resource availability in the genus *Helianthus*. *Helianthus* (sunflowers) is a diverse assemblage of approximately 50 herbaceous annual and perennial dicots. Members of the genus are found in a wide variety of habitats in North America (e.g., deserts, pine savannas, grassland prairies, wetlands) and exhibit relatively high morphological diversity for a single genus, making *Helianthus* well suited for investigations of trait evolution and its relation to habitat resource availability (Heiser et al. 1969; Kane et al. 2013; Donovan et al. 2014; Mason and Donovan 2015). In addition, a recent common garden study of *Helianthus* found that covariation among leaf traits supported a resource acquisition–conservation tradeoff (Mason and Donovan 2015), providing a unique opportunity to assess whether analogous tradeoffs exist in roots of this diverse genus.

We examined genetic differentiation for 12 root morphological (SRL and RTD), chemical (root N and C:N ratio), and anatomical (xylem vessel numbers and cross‐sectional areas) traits across populations of 26 *Helianthus* species (and sister species *Phoebanthus tenuifolius*) in a greenhouse common garden. First, we tested whether there is support for covariation of fine root traits in *Helianthus*. We expected high SRL and root N would be associated with low RTD. We also expected anatomical traits reflecting investment in secondary cell walls (e.g., high xylem vessel number and proportional allocation to xylem and stele) would be associated with high RTD. Strong covariation among traits would suggest the existence of root trait strategies and tradeoffs among root traits. Second, we tested whether root traits were associated with native environment characteristics. We expected populations exhibiting high SRL and root N, and low RTD, would be associated with higher native site resource availability, suggesting repeated evolution (and thus the adaptive value) of a rapid resource acquisition strategy in such sites. Third, we tested whether fine root traits covaried with resource‐use traits in leaves that have been previously reported in a parallel common garden study of *Helianthus* (Mason and Donovan 2015). We expected covariation among fine root and leaf traits would provide evidence for integrated whole‐plant resource‐use strategies, such that populations with root traits suited for rapid resource acquisition and use (high SRL, root N, low RTD) would exhibit analogous leaf traits (high metabolic rates and nutrient concentrations, low structural investment, and short life span).

## Materials and Methods

### Plant material and native habitat characterization

Seeds were collected from one to three populations of each of 26 *Helianthus* species (nine annual species and 15 perennial species; in total, roughly half of the species in genus *Helianthus*) either directly from wild populations or from accessions held by the USDA Germplasm Resources Information Network (USDA National Genetic Resources Program; see geographic locations and accession numbers in Data S1) between 2007 and 2013. *Phoebanthus tenuifolius* seeds were also collected for inclusion in the study as an outgroup for *Helianthus* (Mason and Donovan 2015; Stephens et al. 2015). Of the 58 populations collected in total, 48 were directly collected from wild populations, while 10 were obtained from USDA accessions (nine of which were also directly collected from wild populations; i.e., they had not yet been regrown to maintain seed stocks). Although one to three populations per species may not capture the full range of variation within each species, we chose geographically distant populations across the geographic range of each species to the greatest extent possible in order to maximize the intraspecific variation captured. To characterize the native site of each population seed source, soil and climate data were collected for each site and are reported in a separate study (Mason and Donovan 2015). Five soil cores (5 cm diameter, 0–20 cm depth) were collected representatively across the native site of each population and analyzed by A & L Laboratories (North Chesterfield, VA) for fertility characteristics, and a subsample of each core was analyzed for total N concentration and C:N ratio (Micro‐Dumas combustion; NA1500, Carlo Erba Strumentazione, Milan, Italy) by the Stable Isotope Laboratory at the University of Georgia. Climate data from each seed collection site were extracted from the WorldClim database (Hijmans et al. 2005).

### Experimental design and growth conditions

Due to the large number of plants in this study, the 27 species were divided into two subsets and grown in either summer 2012 or 2013 in the UGA greenhouse facility. Each year, the initial experimental design was a randomized block design to account for potential environmental heterogeneity within the greenhouse, with eight blocks and one replicate plant per population per block. Due to seedling mortality, replicates of several populations were regrown in summer 2014 (see Data S2 for years that replicates of each population were grown). In addition, three populations of *H. annuus* were regrown in each of the 3 years to evaluate and correct for potential variation across years (Mason and Donovan 2015). Each year of the study, seeds were germinated in late May. The blunt end of each seed was scarified and seeds were placed on moist filter paper in petri dishes in darkness for 24 h at room temperature (20°C). Seed coats were removed with forceps, and petri dishes were moved to a controlled environment growth chamber (Conviron, Winnipeg, Canada) set to a 12‐h 25/20°C day/night cycle with 70% relative humidity. Seedlings were misted daily with deionized water for 5 days. Seedlings were then individually transplanted to 7.5 cm deep plugs filled with sand and fertilized daily with a complete nutrient solution (Jack's 20‐10‐20; JR Peters, Inc., Allentown, PA) to allow establishment. One week later, seedlings were transplanted to 22 cm deep Treepots (2.2 L volume; Stuewe and Sons Inc, Corvallis, OR) filled with a 3:1 mix of sand:Turface (fritted clay; Profile Products, Buffalo Grove, IL) and moved to the greenhouse. Plants were watered to field capacity daily, and 5 g of Osmocote Plus 15‐9‐12 slow‐release fertilizer with micronutrients (Scotts, Marysville, OH) was added to the soil surface in each pot. Greenhouse temperature controls were set to maintain temperatures between 18 and 24°C; however, greenhouse temperatures likely deviated above that range given the high summer temperatures during each year of the study. Plants received ambient photoperiod and relative humidity.

### Fine root trait measurements

Each year of the study, root tissue collection took place over 4 days in late July (8 weeks after germination), with two complete blocks harvested per day. Plants were removed from the pots and soil was gently brushed from the root system. Studies have demonstrated that root structure varies with root order, such that first‐ and second‐order roots (the two most distal branching orders; designated here as the fine roots) are generally expected to be the most important for resource uptake (Pregitzer et al. 2002; Guo et al. 2008). To ensure comparison of functionally analogous root tissues across species, a random subsample of first‐ and second‐order roots (following Pregitzer et al. 2002) was collected for each individual plant and placed in plastic bags in a cooler before analysis in the laboratory (Comas and Eissenstat 2004).

Fine root samples were rinsed in deionized water and a single 5 mm segment 2.0 cm from the root tip was cut from a random first‐order root of each sample for anatomical analysis (Hummel et al. 2007). Following removal of the 5 mm segment for anatomical analysis, the remainder of each fine root sample was then separated into two representative subsamples. One subsample, to be used for morphological analysis (SRL and RTD), was stained for 3 min. with 0.01% (w/v) Toluidine Blue O (Carolina Biological Supply, Burlington, NC), spread in a thin layer of water in a clear plastic tray to minimize overlap, and imaged with a desktop scanner at a resolution of 400 dpi. Length and volume for each sample were determined using the software winRHIZO (v. 2002c; Regent Instruments, Quebec, Canada). As root diameter distributions exhibited a left‐skewed distribution, root volume was calculated by the sum of the volumes of each diameter class, rather than by mean diameter (Ryser 2006). Scanned root samples were dried in a forced air drying oven at 60°C for 72 h and weighed. Specific root length was calculated for each sample from total root length and dry mass, and RTD was calculated from dry mass and volume. The second (unstained) fine root subsample was dried at 60°C for 72 h then ground to a fine powder, and root chemistry traits (root N and C:N) were determined by Micro‐Dumas combustion (NA1500, Carlo Erba Strumentazione, Milan, Italy) by the Stable Isotope Laboratory at the University of Georgia. Due to insufficient root material for chemical analyses in several populations, root N and C:N were not assessed in six of the 58 total populations. This led to the exclusion of one species, *H. niveus* ssp. *tephrodes*, from the chemical analyses.

Preparation of samples for anatomical analyses was modified from Freshour et al. (1996). Briefly, the 5 mm fine root segments were fixed in 4% glutaraldehyde buffered 1:1 (v/v) with potassium phosphate buffer (51% 0.1 mol·L^−1^ KH_2_PO_4_ and 49% 0.1 mol·L^−1^ K_2_HPO_4_), moved through an ethanol dehydration series (10%, 25%, 50%, 75%, 95%, 100%, 100%, 100% ethanol; 10 min each), and embedded in increasing concentrations of LR White acrylic resin (Electron Microscopy Sciences, Hatfield, PA) (resin/ethanol: 25/75%, 50/50%, 75/25%, 100/0%, 100/0%; 8 h each). Samples were sliced to 1.0 *μ*m thick sections with an ultramicrotome (Ultracut E, Reichert‐Jung, Vienna, Austria), and stained with Toluidine Blue O (0.1% w/v). Sections were photographed under light microscopy (Axioskop 2, Zeiss, Oberkochen, Germany) and analyzed with ZEN software (Zeiss, Oberkochen, Germany). Root anatomical traits [root cross‐sectional area (CSA), total xylem CSA, number of xylem vessels, number of large xylem vessels (>225 *μ*m^2^), mean vessel CSA, proportion of root CSA in xylem, proportion of root CSA in stele, and number of vessels per unit root CSA] were measured by tracing the appropriate structures with the cursor. Similar to root chemistry, four of the 58 populations were not assessed for anatomical traits due to insufficient root material.

### Data analysis

For populations grown in multiple years, replicate seedlings were pooled across years, and population‐level least‐squares means were calculated for each root trait for all populations (as described below) to account for variation across years. For each class of traits (morphological, chemical, and anatomical), there were a minimum of three replicate seedlings per population, with the following mean number of replicate seedlings (± standard deviation) per population: 9.3 (± 4.9) for morphological analyses, 7.7 (± 4.1) for chemical analyses, and 7.1 (± 3.9) for anatomical analyses.

To calculate population‐level trait means which account for “year” and “block” effects, population least‐squares means were calculated for SRL, RTD, and root N using ANOVA, with population, year, block, and interactions as explanatory variables. Due to logistical constraints, root subsamples assessed for anatomical characteristics were bulked by population; thus, “block” was not recorded for root anatomy data. However, least‐squares means with and without inclusion of block as an explanatory variable were highly correlated for SRL, RTD, and root N, (all *r* ^2^ > 0.98; *P* < 0.001), as were population rankings using Spearman rank correlation (all ρ 0.98; *P* < 0.001). Therefore, block effects were not considered further, and we assessed least‐squares means for all traits using population and year as explanatory variables in ANOVA.

We also calculated phylogenetic signal for each class of traits and habitat variables (root morphology, chemistry, and anatomy, soil, altitude, and climate) using both a multivariate extension of Blomberg's K (Blomberg et al. 2003) described by Adams (2014), as well as Pagel's *λ* (Pagel 1999). Given that several of the traits in our study are mathematically related (e.g., several of the anatomical traits were assessed both individually, as well as in proportions of total root cross‐sectional area), phylogenetic signal was calculated for class of traits rather than individual traits (Adams 2014). Although this procedure assumes that the traits within a class have similar phylogenetic signal, this procedure preserves statistical power by providing broad consensus estimates of phylogenetic signal for classes of traits (Adams 2014). It is also important to note that this approach does not assume that traits within a class are correlated, but rather just that there is a similar evolutionary rate for all the traits within a class. To test for phylogenetic signal, we used the most recent diploid phylogeny of the genus (Stephens et al. 2015). Multivariate Blomberg's K was estimated for each class of traits and habitat variables using the *physignal* function in the R package *geomorph* (Adams and Otárola‐Castillo 2013) in R (R Development Core Team 2008). This procedure tests for significant phylogenetic signal for each class of traits via phylogenetic permutation, in which the observed phylogenetic signal is compared to the phylogenetic signal calculated when trait values have been randomly reshuffled across the tree (Blomberg et al. 2003; Adams 2014). We also used restricted maximum likelihood using the R package *Rphylopars* (Goolsby et al. 2015) to assess Pagel's *λ* of phylogenetic residuals (Revell 2010) for each class of traits, and likelihood ratio tests were performed to test the null hypothesis that error is distributed independently of phylogeny. Failure to reject the null hypothesis of independently distributed error would suggest that phylogenetic correction is statistically inappropriate (Revell 2010).

Pairwise relationships among population‐level root trait least‐squares means and environmental characteristics were assessed using JMP Pro v. 11 (SAS Institute Inc., Cary, NC). We also assessed pairwise relationships among root traits measured in the present study with population values for leaf traits previously reported for a parallel common garden study of *Helianthus* (Mason and Donovan 2015). Both the present study and the Mason and Donovan study examined the same populations of *Helianthus* using the same bulked seed sources to represent each population, and similar plant growth conditions. Although leaf traits were collected according to plant developmental stage (rather than plant age) in the Mason and Donovan study, trait measurements in that study ranged from 6 to 11 weeks postgermination, similar to the present study, in which we assessed root traits at 8 weeks postgermination. We focused on six focal leaf traits reported in regional and global studies of leaf trait covariation which together reflect leaf physiology, structure, and longevity (Reich et al. 1997; Wright et al. 2004; Reich 2014): photosynthesis and respiration per unit mass (A_mass_ and R_mass_, respectively), leaf mass per area (LMA), leaf nitrogen and phosphorus concentrations (leaf N and leaf P, respectively), and leaf life span (LL). Fine root, leaf, and environmental variables were transformed as needed to better approximate assumptions of normality and homoscedasticity. For several root traits, leaf traits, or environmental characteristics, one to two populations were extreme outliers (greater than three standard deviations from the mean; denoted in Data S3). As such, we present pairwise relationships both including and excluding these populations. *R* ^2^ values were considered significant at *P *<* *0.05. Although a *P*‐value correction for multiple statistical tests could potentially reduce type I error rates, these are rarely incorporated in studies examining trait covariation (e.g., Wright et al. 2004; Tjoelker et al. 2005; Comas and Eissenstat 2009; Kembel and Cahill 2011; Chen et al. 2013; Kong et al. 2014), given that *R* ^2^ values allow for biological interpretation of trait relationships, regardless of statistical significance (*sensu* Poorter et al. 2014).

To examine the potential for multitrait axes summarizing covariation in fine root and leaf traits, we conducted a principal component analysis (PCA) incorporating all fine root traits assessed, as well as a PCA incorporating all fine root traits and the six focal leaf traits from Mason and Donovan (2015), using JMP Pro v. 11 (SAS Institute Inc., Cary, NC). We also conducted two additional PCA analyses: one summarizing covariation in fine root anatomical traits and one summarizing covariation in the six focal leaf traits. Principal component plots and loading scores were extremely congruent whether or not statistical outliers (described above) were included in the analyses; thus, we present all PCA results including these outliers.

## Results

### Phylogenetic signal

Multivariate Blomberg's K was nonsignificant for each class of root trait and native site variables (Table 1), suggesting a lack of phylogenetic signal in the data (Blomberg et al. 2003; Adams 2014). Additionally, no evidence for phylogenetic signal was found in the residuals of root morphology, anatomy, soil characteristics, altitude, or climate (Table 1). Because the error in all but one class of traits appears to be distributed independently of phylogeny, phylogenetic correction is statistically inappropriate (Revell 2010). Accordingly, we assumed error was independently distributed for the remainder of analyses.

**Table 1:** Multivariate K‐statistics (Adams 2014) and Pagel's *λ* (Pagel 1999) values of phylogenetic residuals, for each class of root traits and native site characteristics. Root traits and native site characteristics included within each class of variables are described in the text. Statistically significant values (*P* < 0.05) are in boldface type

Variable class	Multivariate K	Pagel's *λ*
Root morphology	0.651	0.134
Root chemistry	1.174	**0.917**
Root anatomy	0.627	0.525
Native site soils	0.675	0.021
Native site altitude	0.845	1.000
Native site climate	0.947	0.401

### Fine root trait–trait relationships

Across populations of 26 *Helianthus* species and *P. tenuifolius*, least‐squares means for root morphological and chemical traits varied roughly twofold, with coefficients of variation between 0.14 and 0.24 for SRL, RTD, root N, and root C:N. Least‐squares means for root anatomical traits varied from twofold to eightfold across populations, with coefficients of variation ranging from 0.30 to 0.72 (Table 2, Data S3).

**Table 2:** Summary statistics of fine root trait least‐squares means among populations of 26 *Helianthus* species and *Phoebanthus tenuifolius*. Specific root length (SRL); root tissue density (RTD); root nitrogen concentration (root N); root carbon:nitrogen ratio (root C:N); root cross‐sectional area (root CSA); total xylem CSA (xylem CSA); number of vessels (no. vess.); number of large vessels (>225 *μ*m^2^; large vess.); mean xylem vessel CSA (mean vessel CSA); proportion of root CSA in xylem (xylem/root CSA); proportion of root CSA in stele (stele/root CSA); number of vessels per unit root CSA (vess./root CSA); coefficient of variation (CV)

Variable class	Root traits	Units	Minimum	Maximum	Mean	CV
Root morphology	SRL	m·g^−1^	161.6	421.2	277.0	0.19
	RTD	g·cm^−3^	0.02	0.05	0.03	0.14
Root chemistry	Root N	g·g^−1^	2.20	5.58	3.50	0.21
	Root C:N	–	6.20	21.43	12.53	0.24
Root anatomy	Root CSA	*μ*m^2^	1.01 × 10^5^	3.95 × 10^5^	2.15 × 10^5^	0.32
	Xylem CSA	*μ*m^2^	309.0	7296.1	1740.0	0.70
	No. Vess.	count	1.08	8.48	3.99	0.34
	Large Vess.	Count	1.00	3.80	2.26	0.30
	Mean Vessel CSA	*μ*m^2^	142.1	1430.8	407.2	0.46
	Xylem/Root CSA	–	0.002	0.039	0.009	0.72
	Stele/Root CSA	–	0.03	0.10	0.05	0.33
	Vess./Root CSA	–	2.97 × 10^−06^	4.79 × 10^−05^	2.19 × 10^−05^	0.43

Among population‐level trait means for morphological and chemical traits, high SRL was associated with low RTD, although neither trait was related to root N or root C:N (Table 3). In terms of the anatomical characteristics underlying these traits, higher SRL was associated with lower root CSA and number of large vessels, but no anatomical traits were related to RTD (Table 3). Root N, on the other hand, was positively associated with root CSA and negatively associated with most other anatomical traits (xylem CSA, number of vessels, proportion of root CSA in xylem and stele, and number of vessels per unit root CSA; Table 3). Associations among anatomical traits were generally stronger than those involving morphological and chemical traits, potentially due in part to the nonindependence of several traits (e.g., number of xylem vessels and number of xylem vessels per unit root CSA). Total xylem CSA and number of large diameter vessels were associated with mean vessel CSA and the proportions of root CSA in xylem and stele, while number of xylem vessels was positively associated with number of large vessels and the proportions of root CSA in xylem and stele (Table 3).

**Table 3:** Significant (*P* < 0.05) *R* ^2^ values among root morphological, chemical, and anatomical traits across populations of 26 *Helianthus* species and *Phoebanthus tenuifolius*. Directionality is indicated in parentheses, with *n* = 52–58 as described in main text. Not significant (ns). Trait abbreviations and units as in Table 2

Root traits	RTD	Root N	Root C:N	Root CSA	Xylem CSA	No. Vess.	Large Vess.	Mean Vessel CSA	Xylem/Root CSA	Stele/Root CSA	Vess./Root CSA
SRL	(−)0.17	ns	ns	(−)0.10	ns	ns	(−)0.09	ns	ns	ns	ns
RTD		ns	ns	ns	ns	ns	ns	ns	ns	ns	ns
Root N			(−)0.91	(+)0.10	(−)0.19	(−)0.20	ns	ns	(−)0.28	(−)0.25	(−)0.27
Root C:N				(−)0.09	(+)0.21	(+)0.19	ns	ns	(+)0.28	(+)0.26	(+)0.27
Root CSA					ns	ns	ns	ns	(−)0.22	(−)0.13	(−)0.44
Xylem CSA						(+)0.34	(+)0.67	(+)0.67	(+)0.75	(+)0.61	(+)0.21
No. Vess.							(+)0.20	ns	(+)0.39	(+)0.35	(+)0.71
Large Vess.								(+)0.48	(+)0.34	(+)0.28	ns
Mean Vessel CSA									(+)0.46	(+)0.35	ns
Xylem/Root CSA										(+)0.82	(+)0.52
Stele/Root CSA											(+)0.44

**Figure 1:**
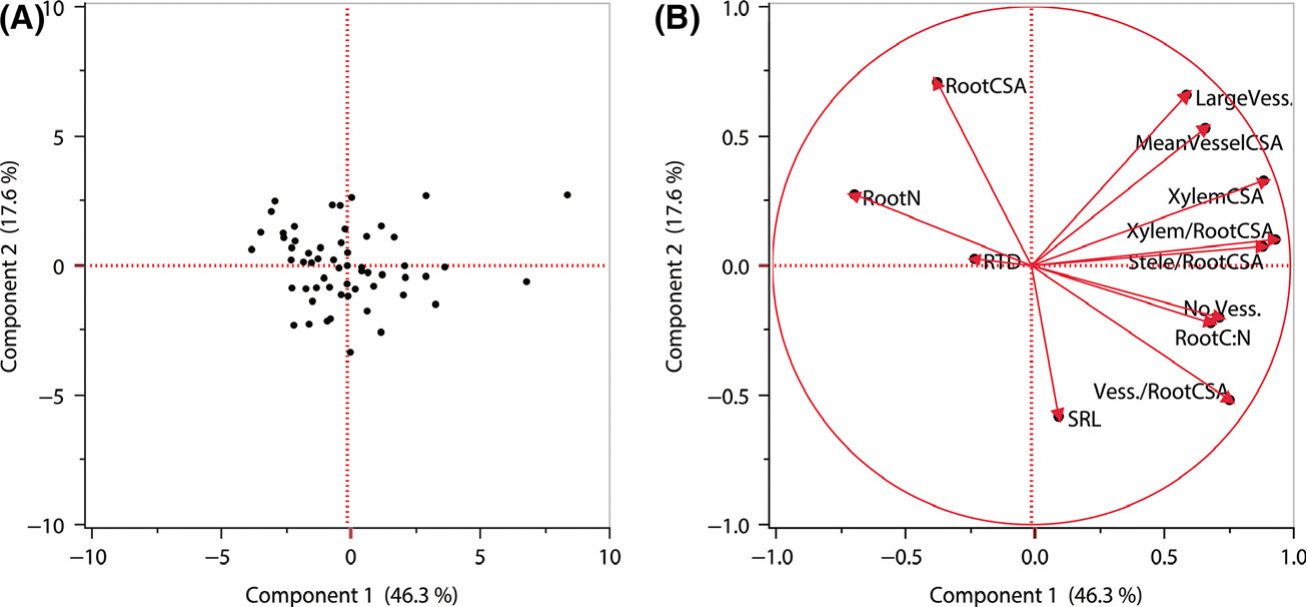
Principal component analysis of 12 fine root traits among populations of 26 *Helianthus* species and *Phoebanthus tenuifolius*. (A) Population distribution along the first two principal component axes; (B) trait loadings biplot. Trait abbreviations and units as in Table 2. See Table 4 for PCA loadings.

**Figure 2:**
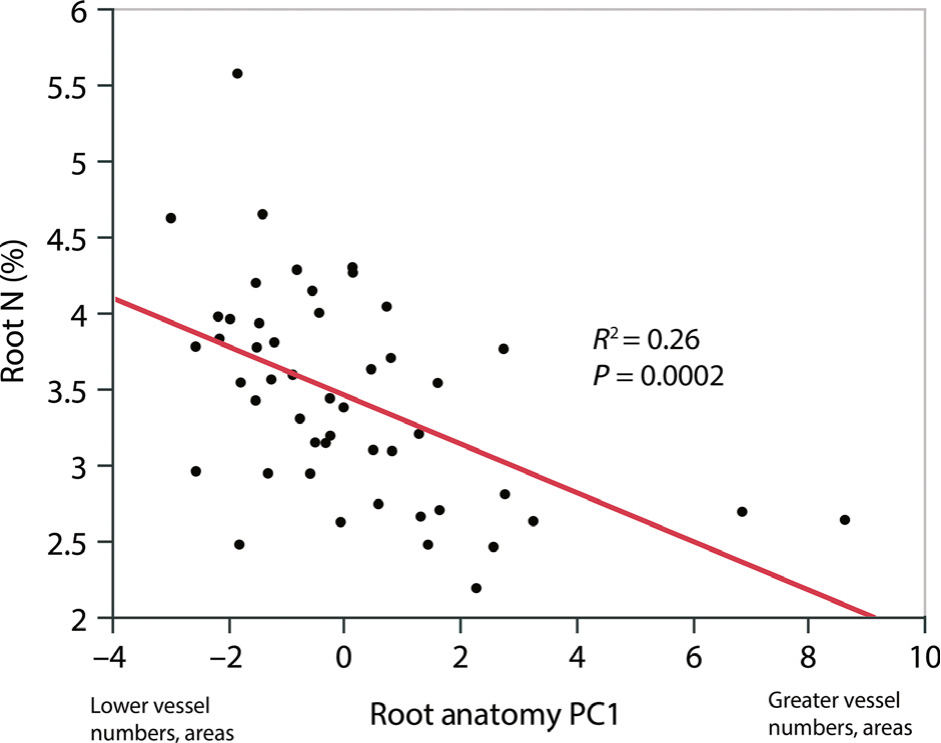
Scatterplot of fine root nitrogen concentration (root N) versus the principal component scores from the first axis of a PCA of fine root anatomical traits (Root Anatomy PC1) in populations of 26 *Helianthus* species and *Phoebanthus tenuifolius*. Higher Root Anatomy PC1 scores indicate a greater number of xylem vessels and larger vessel cross‐sectional areas (see Table S1 for PCA loadings).

These pairwise trait relationships were largely supported by principal component analyses (PCA). In a PCA which included all 12 fine root traits measured, the first PC axis explained nearly half (46.3%) of the variance in fine root traits and primarily revealed a tradeoff between root N (strongly negatively loaded on this axis) and anatomical traits describing xylem vessel number and cross‐sectional area (strongly positively loaded on this axis) (Fig. 1, Table 4). In a PCA including only root anatomical traits, the first axis (Root Anatomy PC1) explained nearly 60% of the variance in root anatomy, and all traits describing xylem vessel numbers and cross‐sectional areas had strong positive loadings on this axis (Table S1). In addition, Root Anatomy PC1 scores were negatively correlated with root N, indicating that greater vessel numbers and cross‐sectional areas were associated with lower root N (Fig. 2).

**Table 4:** Loading scores of fine root traits on the first, second, and third principal component (PC) axes of the principal component analysis depicted in Figure 1. Root trait abbreviations and units as in Table 2

Root Traits	PC Axis 1 (46.3%)	PC Axis 2 (17.6%)	PC Axis 3 (12.3%)
SRL	0.11	**−0.58**	**0.58**
RTD	−0.22	0.03	**−0.80**
Root N	**−0.68**	0.28	0.39
Root C:N	**0.69**	−0.22	−0.48
Root CSA	−0.36	**0.71**	−0.01
Xylem CSA	**0.90**	0.33	0.16
No. Vess.	**0.73**	−0.20	−0.11
Large Vess.	**0.60**	**0.66**	−0.08
Mean Vessel CSA	**0.67**	**0.53**	0.16
Xylem/Root CSA	**0.94**	0.10	0.17
Stele/Root CSA	**0.89**	0.07	0.11
Vess./Root CSA	**0.77**	**−0.52**	−0.03

Strong loadings (>0.49) are bolded.

### Fine root trait–environment relationships

In addition to root morphological, chemical, and anatomical traits, there was substantial variation among populations in characteristics of their native environments. Among soil characteristics, native sites varied from two‐ to fourfold in soil pH and C:N, and varied greater than an order of magnitude in soil N, OM, CEC, K, Mg, and Ca (Data S4; Mason and Donovan 2015). Among climate variables, mean annual temperature ranged from 5.1 to 22.9°C, and mean annual precipitation ranged from 60 to 1839 cm·year^−1^, while native site altitude ranged from 1 to 2566 m above sea level (Data S4; Mason and Donovan 2015).

In general, trait–environment relationships were weak. Lower SRL (and higher RTD) was associated with higher soil CEC, Mg, Ca, and pH (Table 5). Higher root N was associated with lower soil P, but with higher precipitation of the driest month and precipitation of the warmest quarter (Tables 5 and 6). Among anatomical traits, number of xylem vessels was positively associated with soil P, K, Mg, Ca, pH, and CEC (Table 5). Number of vessels, number of large vessels, proportion of root CSA in xylem, and number of vessels per root CSA all decreased with precipitation of the driest month and precipitation of the warmest quarter (Table 6).

**Table 5:** Significant (*P* < 0.05) *r* ^2^ values between root traits and native site soil characteristics across populations of 26 *Helianthus* species and *Phoebanthus tenuifolius*. Directionality indicated in parentheses, with *n* = 52–58 as described in main text. *R* ^2^ values which became nonsignificant when outlier populations were excluded are indicated by (*δ*), and those which became significant when outlier populations were excluded are indicated by (§). Organic matter (OM), soil nitrogen concentration (N), soil carbon:nitrogen (C:N), available phosphorus (P), cation exchange capacity (CEC), exchangeable potassium (K), magnesium (Mg), and calcium (Ca). Not significant (ns). Root trait abbreviations and units as in Table 2

Root traits	Native soil characteristics								
	OM (%)	N (%)	C:N	P (ppm)	CEC (meq/g)	K (ppm)	Mg (ppm)	Ca (ppm)	pH
SRL	ns	(+)0.08	ns	ns	(−)0.15	ns	(−)0.21	(−)0.15	(−)0.07
RTD	ns	ns	ns	ns	(+)0.10	(+)0.09^§^	(+)0.09	(+)0.09	(+)0.09
Root N	ns	ns	ns	(−)0.12	ns	ns	ns	ns	ns
Root C:N	ns	(+)0.10	ns	(+)0.13	ns	(+)0.10	ns	ns	ns
Root CSA	ns	ns	ns	ns	ns	ns	ns	ns	ns
Xylem CSA	ns	ns	ns	(+)0.08^*δ*^	ns	ns	ns	ns	ns
No. Vess.	ns	ns	ns	(+)0.15	(+)0.08	(+)0.12	(+)0.09	(+)0.09	(+)0.09
Large Vess.	ns	ns	ns	ns	(+)0.13	ns	(+)0.11	(+)0.11	ns
Mean Vess. CSA	ns	ns	ns	ns	ns	ns	ns	ns	ns
Xylem/Root CSA	ns	ns	ns	ns	ns	ns	ns	ns	ns
Stele/Root CSA	ns	ns	ns	(+)0.08^*δ*^	ns	(+)0.08^*δ*^	ns	ns	ns
Vess./Root CSA	ns	ns	ns	(+)0.10	ns	(+)0.08^§^	ns	ns	ns

**Table 6:** Significant (*P* < 0.05) *R* ^2^ values between root traits and native climate characteristics and altitude across populations of 26 *Helianthus* species and *Phoebanthus tenuifolius*. Directionality indicated in parentheses, with *n* = 52–58 as described in main text. *R* ^2^ values which became nonsignificant when outlier populations were excluded are indicated by (*δ*). Altitude (Alt.); mean annual temperature (MAT); mean annual precipitation (MAP); precipitation of the driest month (PDM); precipitation of the warmest quarter (PWQ). Not significant (ns). Root trait abbreviations and units as in Table 2

Root traits	Native altitude and climate				
	Alt. (m)	MAT (°C)	MAP (mm)	PDM (mm)	PWQ (mm)
SRL	ns	ns	ns	ns	ns
RTD	ns	ns	ns	ns	ns
Root N	ns	ns	ns	(+)0.11	(+)0.14
Root C:N	ns	ns	ns	(−)0.11	(−)0.20
Root CSA	ns	ns	ns	ns	ns
Xylem CSA	ns	ns	ns	(−)0.09^*δ*^	(−)0.08^*δ*^
No. Vess.	ns	(−)0.16	ns	(−)0.19	(−)0.14
Large Vess.	ns	ns	ns	(−)0.11	(−)0.11
Mean Vess. CSA	ns	ns	ns	ns	ns
Xylem/Root CSA	ns	ns	ns	(−)0.09^*δ*^	(−)0.13
Stele/Root CSA	(+)0.14	(−)0.08^*δ*^	ns	ns	(−)0.15
Vess./Root CSA	(+)0.08	(−)0.12	ns	(−)0.08	(−)0.11

### Fine root and leaf trait relationships

**Figure 3:**
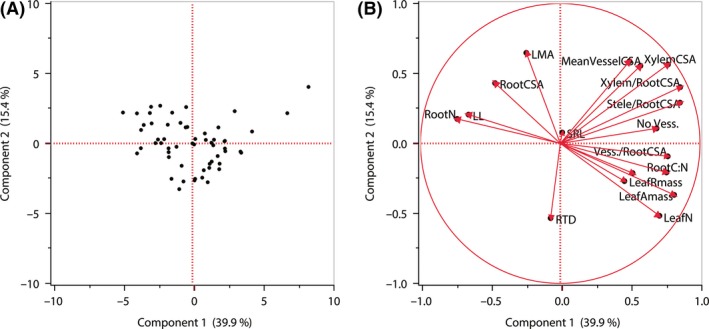
Principal component analysis of 12 fine root traits and six leaf traits among populations of 26 *Helianthus* species and *Phoebanthus tenuifolius*. (A) Population distribution along the first two principal component axes; (B) trait loadings biplot. Root trait abbreviations as in Table 2; leaf trait abbreviations as in the main text (see Table S2 for PCA loadings).

**Figure 4:**
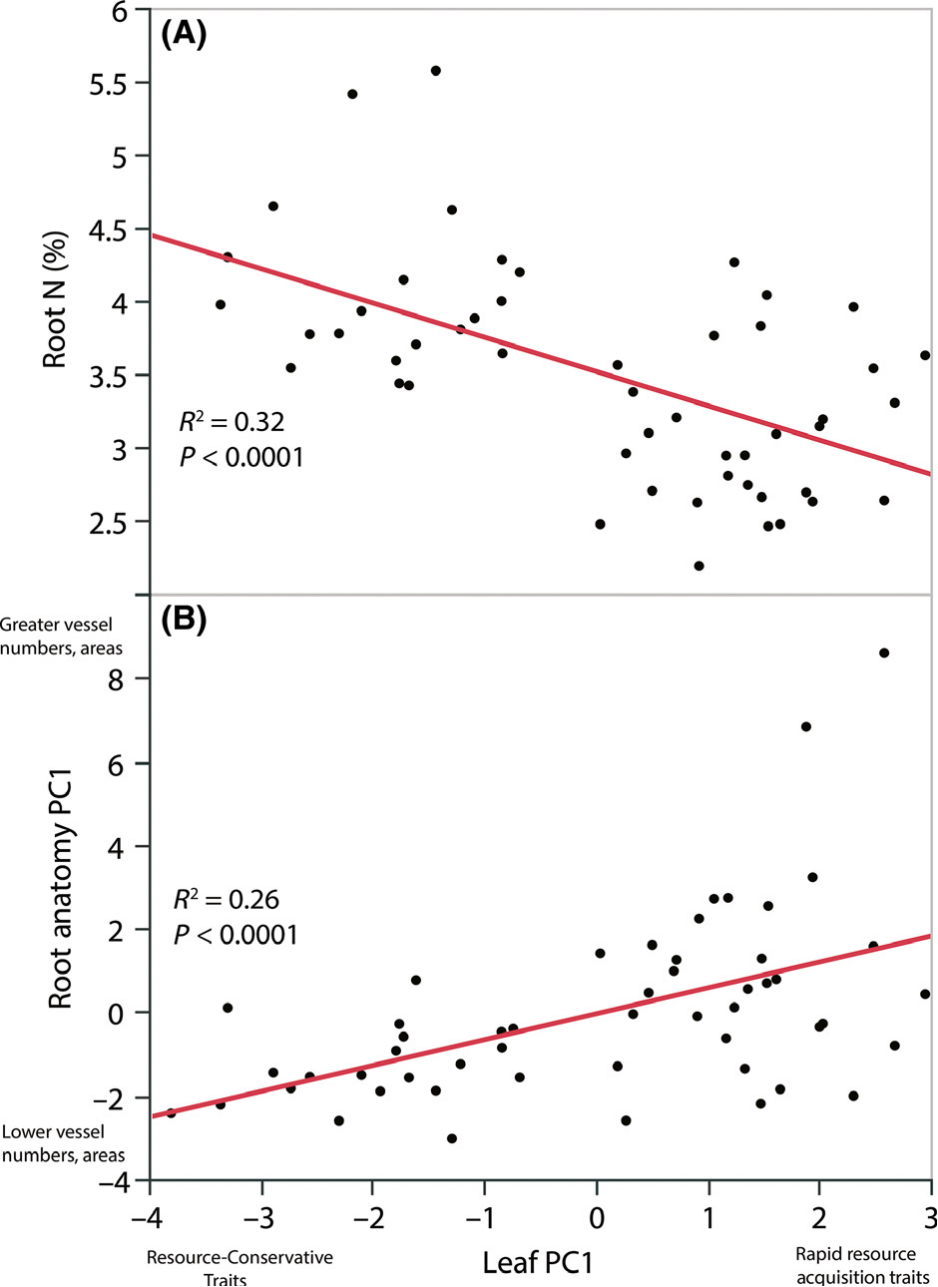
Scatterplots of: (A) fine root nitrogen concentration (root N) versus the principal component scores from the first axis of a PCA of leaf traits (Leaf PC1; based on leaf traits reported in Mason and Donovan 2015), and (B) principal component scores from the first axis of a PCA of fine root anatomical traits (Root Anatomy PC1) versus Leaf PC1, in populations of 26 *Helianthus* species and *Phoebanthus tenuifolius*. Higher Leaf PC1 scores indicate rapid resource acquisition and use traits (higher Leaf A_mass_, R_mass_, Leaf N, and Leaf P, and lower LMA and LL; see Table S2 for PCA loadings), while higher Root Anatomy PC1 scores indicate a greater number of xylem vessels and larger vessel cross‐sectional areas (see Table S1 for PCA loadings).

We also tested for associations between fine root traits assessed in the present study and leaf traits reported from a parallel common garden study (Mason and Donovan 2015) of the same *Helianthus* populations. In pairwise comparisons, high leaf A_mass_ and leaf N were associated with lower root N and CSA, but with higher values of anatomical traits describing water transport capacity, including number of xylem vessels, proportion of root CSA in xylem, and number of vessels per root CSA (Table 7). Leaf life span exhibited the opposite associations with root traits, being positively associated with higher root N and CSA, and negatively with anatomical traits describing water transport capacity (Table 7). As with fine root traits, these fine root–leaf trait relationships were largely supported by PCA. The first axis of a PCA which incorporated all fine root and leaf traits measured explained nearly 40% of the variation in these traits and primarily indicated that high leaf A_mass_ and leaf N were associated with greater xylem vessel numbers and cross‐sectional areas, as well as with low root N (Fig. 3, Table 8). In a PCA including only leaf traits, the first principal component axis (Leaf PC1) explained over half (54%) of the variance among the leaf traits (Table S2). Higher Leaf PC1 scores (indicating high A_mass_, R_mass_, Leaf N, and Leaf P, but low LMA and short LL) were associated with lower root N (Fig. 4A). However, higher Leaf PC1 scores were associated with higher Root Anatomy PC1 scores (indicating greater vessel numbers and cross‐sectional areas; Fig. 4B).

**Table 7:** Significant (*P* < 0.05) *R* ^2^ values between root traits measured in the present study, and leaf traits reported in a parallel study (Mason and Donovan 2015) across populations of 26 *Helianthus* species and *Phoebanthus tenuifolius*. Directionality indicated in parentheses, with *n* = 52–58 as described in main text. *R* ^2^ values which became nonsignificant when outlier populations were excluded are indicated by (*δ*). Not significant (ns). Root trait abbreviations and units as in Table 2; leaf trait abbreviations as in the main text

Root traits	Leaf traits assessed by Mason and Donovan (2015)					
	Leaf A_mass_ (nmol·CO_2_·g^−1^·s^−1^)	Leaf R_mass_ (nmol·CO_2_·g^−1^·s^−1^)	Leaf N (%)	LL (days)	Leaf P (%)	LMA (g·m^−2^)
SRL	ns	(−)0.10	ns	ns	ns	ns
RTD	ns	ns	ns	ns	ns	(−)0.17
Root N	(−)0.38	ns	(−)0.29	(+)0.35	(−)0.09	ns
Root C:N	(+)0.43	ns	(+)0.31	(−)0.40	(+)0.10	(−)0.12
Root CSA	(−)0.24	(−)0.13	(−)0.27	(+)0.08	(−)0.10	ns
Xylem CSA	(+)0.14^*δ*^	ns	ns	(−)0.16	ns	ns
No. Vess.	(+)0.23	ns	(+)0.15	(−)0.08	ns	ns
Large Vess.	ns	ns	ns	(−)0.08	ns	ns
Mean Vessel CSA	(+)0.08^*δ*^	ns	ns	(−)0.15	ns	ns
Xylem/Root CSA	(+)0.30	(+)0.11	(+)0.18	(−)0.20	(+)0.14	ns
Stele/Root CSA	(+)0.31	(+)0.18	(+)0.24	(−)0.24	(+)0.22	ns
Vess./Root CSA	(+)0.29	(+)0.12	(+)0.28	(−)0.11	(+)0.10	ns

**Table 8:** Loading scores of fine root and leaf traits on the first, second, and third principal component (PC) axes of the principal component analysis depicted in Figure 3. Root trait abbreviations and units as in Table 2; leaf trait abbreviations as in the main text

Root and leaf traits	PC axis 1 (39.9%)	PC axis 2 (15.4%)	PC axis 3 (11%)
SRL	0.01	0.05	**−0.84**
RTD	−0.07	**−0.53**	**0.52**
Root N	**−0.74**	0.18	0.07
Root C:N	**0.76**	−0.21	0.03
Root CSA	−0.47	0.43	**0.51**
Xylem CSA	**0.77**	**0.56**	0.07
No. Vess.	**0.69**	0.11	−0.21
Large Vess.	**0.50**	**0.59**	0.46
Mean Vessel CSA	**0.57**	**0.55**	0.29
Xylem/Root CSA	**0.86**	0.40	−0.08
Stele/Root CSA	**0.86**	0.29	−0.04
Vess./Root CSA	**0.77**	−0.09	−0.46
Leaf A_mass_ (nmol·CO_2_·g^−1^·s^−1^)	**0.81**	−0.37	0.15
Leaf R_mass_ (nmol·CO_2_·g^−1^·s^−1^)	0.46	−0.27	0.21
LMA (g·m^−2^)	−0.24	**0.65**	−0.22
LL (days)	**−0.65**	0.21	−0.18
Leaf N (%)	**0.71**	**−0.52**	0.03
Leaf P (%)	**0.52**	−0.21	0.11

Strong loadings (>0.49) are bolded.

## Discussion

### Fine root trait relationships suggest a tradeoff between root N and water transport

Least‐squares means of fine root morphological, chemical, and anatomical traits varied considerably across populations of 26 *Helianthus* species and sister species *P. tenuifolius*, with variation in SRL and root N comparable to that seen in a study of 65 tree species (representing 51 genera and 30 families) located across a broad environmental gradient in China (Chen et al. 2013). In conjunction with wide variation in trait values, root traits exhibited little phylogenetic signal, suggesting high evolutionary lability of these traits both within and among species (Blomberg et al. 2003; Donovan et al. 2014). Although these findings were perhaps not surprising, given the rapid speciation in this genus and the wide variety of environments inhabited by *Helianthus* (Kamilar and Cooper 2013), high evolutionary trait lability supports this genus as a strong model system for examining how traits evolve with respect to one another and to environmental resource availability. We indeed found evidence for trait covariation and tradeoffs both within fine roots and among roots and leaves in *Helianthus*, but our findings did not always agree with expectations.

SRL, RTD, and root N are among the most commonly measured root traits (Comas et al. 2002; Comas and Eissenstat 2009; Chen et al. 2013; Kong et al. 2014) and are generally hypothesized to covary, with high SRL and root N associated with low RTD (Roumet et al. 2006; Kembel and Cahill 2011; Reich 2014). Although we did find a negative relationship between SRL and RTD, supporting several field studies of herbaceous and woody plants (Comas and Eissenstat 2009; Kembel and Cahill 2011), neither SRL nor RTD was related to root N, suggesting there is no single multitrait axis formed by these traits (Fort et al. 2012; Chen et al. 2013). In terms of anatomical characteristics underlying fine root traits, high SRL was associated with few large vessels, suggesting a potential tradeoff between maximizing soil exploration and hydraulic conductance. However, high SRL was also associated with smaller root CSA (thinner diameter), suggesting that hydraulic conductance could potentially be maximized in high SRL roots by maximizing radial, rather than axial, conductance. In addition, variation in RTD was largely not associated with aspects of internal root anatomy. The lack of strong relationships among SRL, RTD, and anatomical traits suggests that different combinations of anatomical traits can produce high (or low) SRL and RTD in *Helianthus*. This may partially explain why variation in root anatomical traits was generally greater than variation in morphological traits.

High root N was associated with lower values of traits related to xylem anatomy, including total xylem CSA and number of vessels, number of large vessels, proportion of root CSA in xylem and stele, and number of vessels per unit root CSA. This is perhaps not surprising, given the low‐nutrient content of xylem tissues (Li et al. 2010), yet it suggests that high‐nutrient concentrations and high water transport may trade off in *Helianthus*. Although root N per unit length may be similar in roots differing in these anatomical traits, root N per unit mass, which was assessed in the present study, has been associated with metabolic functions such as nutrient uptake rates (Comas et al. 2002), respiration (Tjoelker et al. 2005; Reich et al. 2008), and relative growth rate (Reich et al. 1998). Thus, our findings suggest that fine roots may maximize such metabolic functions at the expense of water transport capacity, regardless of root N per unit length.

### Root–environment associations do not always agree with expectations

Although high SRL is expected to characterize fast‐growing species typical of resource‐rich sites (Reich et al. 1998; Wright and Westoby 1999; Nicotra et al. 2002; Fort et al. 2012), and to allow plants to efficiently explore the soil for nutrients (Ryser 2006), SRL was only weakly positively associated with soil N and negatively with soil Mg and Ca. High RTD was also weakly associated with higher fertility (soil Mg and Ca) contrary to the expectation that it should be characteristic of slow‐growing species typical of resource‐limited sites due to selection for mechanically tough roots suited for resource conservation (Wahl and Ryser 2000; Craine et al. 2001; Fort et al. 2012). Interestingly, weak relationships between SRL and RTD with environmental variables in this study of fine root traits differ from a study of trait–environment relationships at the whole root system level in *Helianthus*. A common garden study of six *Helianthus* species chosen as phylogenetically independent contrasts with respect to native soil fertility found no evidence for adaptive differentiation in whole root system SRL or RTD across species' native sites (Bowsher et al. 2015). This suggests that cross‐species variation in root traits may depend on the level of observation (fine roots vs. the whole root system) in herbaceous species, potentially due to functional differentiation within individual root systems, as has been reported for woody species (Pregitzer et al. 2002; Guo et al. 2008). Additionally, the lack of a relationship between fine root SRL and mean annual precipitation in the present study contrasts with previous reports that species native to high rainfall sites have higher whole root system SRL than those native to low rainfall sites (Wright and Westoby 1999; Nicotra et al. 2002). Again, this may be due to examination of fine roots rather than the whole root system, but may also reflect that the populations used in our study differed in both native site rainfall and soil nutrient availability. Therefore, the lack of a relationship between fine root SRL and mean annual precipitation in our study may reflect interacting selection pressures between rainfall, nutrient availability, or other unmeasured environmental variables (discussed below; Ryser 2006; Comas et al. 2012).

Several anatomical traits involving both xylem vessel size and number were weakly negatively associated with precipitation metrics, suggesting the possibility for high water transport capacity in populations native to extremely dry environments. This may reflect the prevalence of the “live fast, die young” strategy of rapid resource acquisition in water‐limited environments described previously in several annual *Helianthus* species (Ludwig et al. 2004, 2006; Brouillette et al. 2014). Large, numerous vessels may allow such species to quickly complete their life cycles by maximizing water transport during early season episodes of high water availability (Nicotra et al. 2002). Indeed, in a parallel greenhouse study of leaf traits, Mason and Donovan (2015) found that populations with high A_mass_ were associated with drier environments. Thus, high water transport capacity in roots likely serves to fulfill the high transpirational demand required for attaining high A_mass_, thereby supporting a rapidly growing, drought‐escape strategy.

### Leaf and root trait covariation in relation to whole‐plant function

Hypotheses of root trait covariation have largely been based on the tight correlations in leaf structure and physiology seen at a global scale (Reich et al. 1997; Wright et al. 2004). In *Helianthus*, variation in leaf traits generally matches expectations of an acquisition–conservation tradeoff, with high A_mass_ associated with high leaf N, low LMA, and short leaf life span (Table S2; Mason and Donovan 2015). Here, we included the majority of the taxa included in the Mason and Donovan study (58 populations representing 27 species vs. 83 populations representing 28 species) and leveraged both datasets to investigate whether fine root traits covary with resource‐use traits in leaves. Although we found mostly weak support for covariation among fine root traits in our study, several associations between root and leaf traits suggest that some root traits are linked to shoot demands. In particular, rapid resource acquisition traits in leaves (high A_mass_ and leaf N) were associated with greater xylem vessel number and cross‐sectional area, reflecting the high water transport capacity required for high A_mass_. However, in contrast to the positive association between leaf N and root N in meta‐analyses of global tissue nutrient concentrations (Craine et al. 2005), high leaf N and A_mass_ were associated with low root N in our study. This suggests that complex and potentially opposing resource strategies exist across leaves and roots in *Helianthus*. For example, although leaf traits indicative of rapid metabolic rates (high A_mass_, R_mass_, and leaf N, short leaf life span) were associated with fine roots with high capacity for water transport, they were also associated with low root N, suggesting low metabolic rates in roots and longer root life span. Similarly, although resource‐conservative leaf traits related to lower productivity and longer persistence (low A_mass_ and leaf N, longer leaf life span) were associated with low water transport capacity, they were also associated with high root N, suggesting rapid metabolic rates in roots and faster root turnover. Although these findings contradict hypotheses of a unified resource acquisition–conservation tradeoff at the whole‐plant level (Freschet et al. 2010; Reich 2014), these trait combinations may be beneficial in *Helianthus* in particular habitats. For example, while high A_mass_ and leaf N were associated with drier habitats, suggesting a fast‐growing strategy for exploiting brief pulses of water availability, low root N may allow fine roots to persist between those pulses due to low root metabolic rates and slow turnover.

Compared to relationships among leaf traits in *Helianthus* (Mason and Donovan 2015), relationships between fine root traits and between root traits and environmental characteristics were generally weak, likely reflecting the different selection pressures shaping fine roots and leaves (Craine et al. 2005; Withington et al. 2006). While leaves function in the acquisition of carbon and sunlight, which are typically in high abundance in the open habitats of *Helianthus*, roots function in the acquisition of water and numerous nutrients which vary in their mobility, distribution, and temporal availability in soil. Selective pressures to simultaneously balance water and nutrient acquisition along with other belowground functions may even conflict with one another (Ryser 2006). For example, selection for high SRL in low‐nutrient soils to maximize root surface area for nutrient uptake (Paz 2003; Holdaway et al. 2011), but also for low SRL in arid habitats to increase root drought resistance (Nicotra et al. 2002; Ryser 2006; Markesteijn and Poorter 2009), would result in conflicting selection in low‐nutrient, arid habitats. Complex responses to different selective pressures may therefore impede the evolution of a single axis of strategies (from rapid acquisition of all resources, to greater conservation of resources) in roots (Withington et al. 2006), as seen in the apparent tradeoff between nutrient and water relations in *Helianthus*. Additionally, the lack of strong correlations between fine root traits and native habitat characteristics suggests different combinations of root traits may equally optimize resource acquisition and use (Comas et al. 2012). For example, it has been suggested that thin, highly branched roots are effective in acquisition of immobile nutrients such as phosphorus (Brundrett 2002; Holdaway et al. 2011). However, higher arbuscular mycorrhizal colonization rates, which are also linked with higher phosphorus acquisition (Smith and Read 2008; Deguchi et al. 2012), have been found in thicker roots with little branching (Comas et al. 2014; Kong et al. 2014). Clearly, the selection pressures shaping root form and function differ from those of leaves and may partially explain why studies investigating correlations among leaf and root traits have found mixed results (Withington et al. 2006; Zangaro et al. 2008; Kembel and Cahill 2011; Chen et al. 2013).

An important consideration of our study is that many of the root traits we assessed are known to exhibit phenotypic plasticity in response to environmental conditions (Wahl et al. 2001; Espeleta and Donovan 2002; Ryser 2006), and species‐ or trait‐specific responses to the growth conditions of our study could potentially influence the trait relationships detected. For example, a recent study found that species‐specific responses to resource (water and nitrogen) availability resulted in shifts in the strength of leaf trait relationships detected under different treatment conditions (Wright and Sutton‐Grier 2012). However, it is important to note that nonlimiting resource conditions, such as those used in the present study, are generally expected to maximize phenotypic variation among genotypes with contrasting resource‐use strategies (Chapin et al. 1993; Richards et al. 2010). Nevertheless, future common garden studies examining both fine root and leaf trait variation under resource limitations could shed light on the potential impacts of resource availability on trait relationships within and across plant organs.

Overall, our common garden study of genetic differentiation across environments found large variation in fine root traits in *Helianthus*. Although associations among most root traits were generally weak or nonexistent, we provided evidence for a tradeoff in root N and xylem vessel traits, suggesting that roots have either evolved high‐nutrient concentrations, supporting fast metabolic rates, or high xylem vessel numbers and cross‐sectional areas, supporting high water transport capacity. We also showed that leaf traits associated with rapid resource acquisition (A_mass_ and leaf N) were associated with higher xylem vessel numbers and cross‐sectional areas in roots, indicative of high transport capacity needed for high A_mass_. However, high A_mass_ and leaf N were also associated with low root N, suggesting slow root metabolic rates and root turnover, and therefore opposing resource‐use strategies at the root and leaf level. Our findings add to the growing list of both common garden and field studies which report relationships between leaf and fine root traits that are inconsistent with the predictions of an integrated, whole‐plant tradeoff between resource acquisition and conservation (Craine et al. 2001; Craine and Lee 2003; Tjoelker et al. 2005; Withington et al. 2006; Kembel and Cahill 2011). Further studies including additional growth forms and functional types are now needed to disentangle whether the complex trait relationships, such as those seen in *Helianthus*, are indeed the norm in higher plants.

## Conflict of Interest

None declared.

## Supporting information


**Data S1.** Population collection sites and USDA National Genetic Resources Program (GRIN) accession numbers for populations of 26 *Helianthus* species and *Phoebanthus tenuifolius*.

**Data S2.** Number of replicate *Helianthus* and *Phoebanthus tenuifolius* seedlings per population used for analysis of root morphology, chemistry, and anatomy in each year of the study and pooled over the entire study.

**Data S3.** Least‐squares means for fine root traits in populations of 26 *Helianthus* species and *Phoebanthus tenuifolius*.

**Data S4.** Native site characteristics for populations of 26 species of *Helianthus* and *Phoebanthus tenuifolius*, representing a subset of the data reported in Mason and Donovan (2015).

**Table S1.** Loading scores in a principal components analysis of fine root anatomical traits.

**Table S2.** Loading scores in a principal components analysis of leaf traits (leaf trait data from Mason and Donovan 2015).
