# Supplementary material for: Fine root tradeoffs between nitrogen concentration and xylem vessel traits preclude unified whole‐plant resource strategies in Helianthus
Source: Ecol Evol. 2016 Jan 20;6(4):1016–31. doi: 10.1002/ece3.1947 (PMC4761775; doi:10.1002/ece3.1947)
Supplement: Supplementary file 5 — Table S1. Loading scores in a principal components analysis of fine root anatomical traits. [file ECE3-6-1016-s005.docx]

| Root Traits |  | Root Anatomy  PC1 (59.4%) | Root Anatomy  PC2 (22.7%) |
| --- | --- | --- | --- |
| Root CSA |  | -0.33 | 0.79 |
| Xylem CSA |  | 0.94 | 0.25 |
| No. Vess. |  | 0.72 | -0.35 |
| Large Vess. |  | 0.66 | 0.57 |
| Mean Vessel CSA |  | 0.72 | 0.52 |
| Xylem/Root CSA |  | 0.97 | 0.01 |
| Stele/Root CSA |  | 0.91 | 0.02 |
| Vess./Root CSA |  | 0.73 | -0.63 |

**Table S1.** Loading scores on the first and second principal components (PC) axes of a principal components analysis of root anatomical traits. The proportion of variance explained by Root Anatomy PC1 and PC2 are indicated. Root trait abbreviations and units as in Table 2.
