# Supplementary material for: Fine root tradeoffs between nitrogen concentration and xylem vessel traits preclude unified whole‐plant resource strategies in Helianthus
Source: Ecol Evol. 2016 Jan 20;6(4):1016–31. doi: 10.1002/ece3.1947 (PMC4761775; doi:10.1002/ece3.1947)
Supplement: Supplementary file 6 — Table S2. Loading scores in a principal components analysis of leaf traits (leaf trait data from Mason and Donovan 2015). [file ECE3-6-1016-s006.docx]

**Table S2.** Loading scores on the first and second principal components (PC) axes of a principal components analysis of leaf traits (leaf trait data from Mason and Donovan 2015). The proportion of variance explained by Leaf PC1 and PC2 are indicated. Leaf trait abbreviations and units as in the main text.

| Leaf Traits |  | Leaf PC1  (54%) | Leaf PC2  (16.3%) |
| --- | --- | --- | --- |
| Leaf A_mass_ (nmol CO_2_ g^-1^ s^-1^) |  | 0.91 | -0.20 |
| Leaf R_mass_ (nmol CO_2_ g^-1^ s^-1^) |  | 0.63 | 0.52 |
| LMA (g m^-2^) |  | -0.54 | 0.53 |
| LL (days) |  | -0.70 | 0.24 |
| Leaf N (%) |  | 0.90 | -0.06 |
| Leaf P (%) |  | 0.65 | 0.57 |
